# Molecular Characterization of the *env* Gene of Bovine Leukemia Virus in Cattle from Pakistan with NGS-Based Evidence of Virus Heterogeneity

**DOI:** 10.3390/pathogens10070910

**Published:** 2021-07-19

**Authors:** Marzena Rola-Łuszczak, Ali Sakhawat, Aneta Pluta, Anna Ryło, Arkadiusz Bomba, Nazia Bibi, Jacek Kuźmak

**Affiliations:** 1 Department of Biochemistry, National Veterinary Research Institute, 24-100 Puławy, Poland; aneta.pluta@piwet.pulawy.pl (A.P.); anna.rylo@piwet.pulawy.pl (A.R.); jkuzmak@piwet.pulawy.pl (J.K.); 2 National Veterinary Laboratories, Islamabad 45500, Pakistan; sakhawatbalti@yahoo.com; 3 Animal Quarantine Department, Ministry of National Food Security and Research, Peshawar 25000, Pakistan; 4 Department of Bio Sciences, COMSATS University, Islamabad 45500, Pakistan; 5 Department of Omics Analysis, National Veterinary Research Institute, 24-100 Puławy, Poland; arkadiusz.bomba@piwet.pulawy.pl

**Keywords:** bovine leukemia virus (BLV), phylogenetic analysis, genetic variability, quasispecies, Pakistan

## Abstract

Characterization of the global genetic diversity of the bovine leukemia virus (BLV) is an ongoing international research effort. Up to now BLV sequences have been classified into eleven distinct genotypes. Although BLV genotyping and molecular analysis of field isolates were reported in many countries, there is no report describing BLV genotypes present in cattle from Pakistan. In this study we examined 27 *env* gene sequences from BLV-infected cattle coming from four farms located in Khyber Pakhtunkwa, Gilgit Baltisan and Punjab provinces. Phylogenetic analyses revealed the classification of Pakistani sequences into genotypes G1 and G6. The alignment with the FLK-BLV sequence revealed the presence of 45 mutations, namely, seven in genotype G1 and 33 in genotype G6. Five mutations were found in both, G1 and G6 genotypes. Twelve amino acid substitutions were found in the analyzed sequences, of which only one P264S was specific for sequences from Pakistan. Furthermore, a certain degree of nucleotide heterogeneity was identified by NGS. These results highlight the need for further study on the importance of genetic variability of BLV, especially in the context of its pathogenicity and potential effect on serological detection.

## 1. Introduction

Bovine leukemia virus (BLV) belongs to the *Deltaretrovirus* genus of the *Retroviridae* family. The virus is the etiologic agent of enzootic bovine leucosis (EBL), the most frequent and the most important virus-induced tumor disease in cattle [1,2]. BLV causes a lifelong infection characterized by high level of virus cell-association, virus persistence and integration of BLV DNA in provirus form into the host genome [1]. Most of the infected cattle remain clinically healthy and are referred to as aleukemic (AL). After a latency that extends from a few months to several years, about one-third of infected animals develop a polyclonal proliferation of B lymphocytes, mainly CD5^+^ cells, called persistent lymphocytosis (PL). Persistent lymphocytosis is usually stable for several years, but in 1–5% of animals it can progress to the malignant lymphoma, which is the most conspicuous clinical manifestation of BLV infection [3].

Currently, BLV infection has a worldwide distribution and seroepidemiological studies show high prevalence in North and South America, some Asiatic and Middle Eastern countries, and Eastern Europe [4,5].

EBL causes important economic losses in the dairy cattle industry associated with decreased milk production, premature culling, restriction in the export of live animals, and the cost of an eradication campaign [6,7,8,9]. Additionally, BLV infection impairs the immune system and predisposes animals to other diseases [10,11]. Under natural conditions, the virus is mainly transmitted through infected cells, especially by blood and bloodsucking insects. Vertical transmission in utero, or through colostrum and milk, has been documented [12,13].

BLV is classified as a so-called complex retrovirus since its genome contains the *gag*, *pro*, *pol* and *env* genes, which encode structural proteins and enzymes, the regulatory genes *tax* and *rex,* and accessory genes R3 and R4, as well as microRNAs (miRNAs) [1,14]. The *env* gene encodes an envelope glycoprotein consisting of a 51-kDa extracellular surface subunit (SU, gp51) and a 30-kDa transmembrane subunit (TM, gp30). The gp51 surface protein contains the receptor binding domain (RBD), which is indispensable for viral entry into host cells [15,16], and linear and conformational epitopes that are major determinants of virus neutralization and inhibition of syncytium formation [17,18]. Additionally, three neutralization domains, ND1, ND2 and ND3, inducing BLV-neutralizing antibodies were identified [19]. The N-terminal and internal part of gp51 contains the CD4^+^ and CD8^+^ T-cell epitopes, as well as gp51N5, gp51N11 and gp51N12 epitopes, which are an immunologic target for cytotoxic T lymphocytes (CTL) [20]. The glycoprotein gp30 contains an extracellular domain encompassing the hydrophobic fusion peptide and a cytoplasmic domain with a YXXL motif that is supposed to be involved in signal transduction pathways [21].

Over the last two decades, many studies were undertaken to investigate the phylogeny and genetic variability of BLV isolates from different geographical areas [22,23,24,25,26,27,28,29,30]. Almost all of these studies focused primarily on the partial or entire analysis of the *env* gene including gp51 coding sequence, since the gp51 glycoprotein plays an essential and indispensable role in BLV infectivity [15]. Phylogenetic analysis from previous studies grouped *env*-derived sequences into three [31], four [32] and seven distinct genotypes [23]. Further studies showed the existence of genotype 8 in cattle from Croatia [33], genotype 9 in cattle from Bolivia [34] and genotype 10 in Thailand [27] and Myanmar [28]. Finally, the new genotype 11 was identified as the last one in China [35]. All together these studies showed global diversification of BLV isolates grouped into at least 11 genotypes (G1–G11). It has also been revealed that clustering of countries into a particular genotype is generally associated with geographic origin of the isolate [23]. These studies revealed that *env* sequences, representing geographically different isolates, shared high sequence homology among isolates; however, many of them showed the presence of small scale point mutations [4]. It was shown that some of these mutations are associated with infectivity, replication and pathogenesis of BLV [36,37], and can change the serological status of infected cattle [38]. Therefore, the analysis of BLV genetic diversity on a global scale is an ongoing research effort, since it can explain the features of genetic variability of field strains of BLV and its association with disease progression. It can also be helpful to understand shortcoming of diagnostic tests in vitro.

Although BLV genotyping and molecular analysis of field isolates have been reported in many countries through all continents, currently there is no report describing the circulation of BLV genotypes in Pakistan, with their subsequent molecular analysis. Pakistan has a large population of cattle, accounting for close to 50 million heads, which are an important component of the rural economy for dairy and dairy product production [39]. Recent studies showed serological evidence of infection with BLV in cattle from different regions in Pakistan [40,41]. In this study, we report the molecular analysis of BLV sequences obtained from infected cattle from different provinces in Pakistan. We focused on the analysis of *env* gene sequences encoding the complete gp51 surface glycoprotein using PCR amplification and Sanger sequencing. Furthermore, Next Generation Sequencing (NGS) was employed to show the genetic variability of *env* sequence.

## 2. Results

### 2.1. Results of PCR Amplification on Blood and Tissue Samples

Forty DNA samples, including 31 samples prepared from blood and nine prepared from different tissues were tested by nested PCR. In 30 samples prepared from PBLs, 444 bp products were found, and in two samples prepared from different tissues. DNA samples, positive by nested PCR, were then subjected to seminested PCR. In 24 samples from PBLs, 993 bp products were detected, and none in samples from tissues (Table 1). Taking into account the results of both amplifications, sequence analysis was performed on 27 sequences, which included 24 sequences 903 bp long and three sequences 400 bp long. Both 903 bp and 400 bp long fragments were included in the analysis after subtracting the respective length of the primers and additional nucleotides for the 903 bp fragment, to obtain sequence coding signal peptide and gp51protein (Table 1 and Table S1).

### 2.2. Phylogenetic Analysis Based on 400 and 903 bp env Gene Sequences

**Figure 1:**
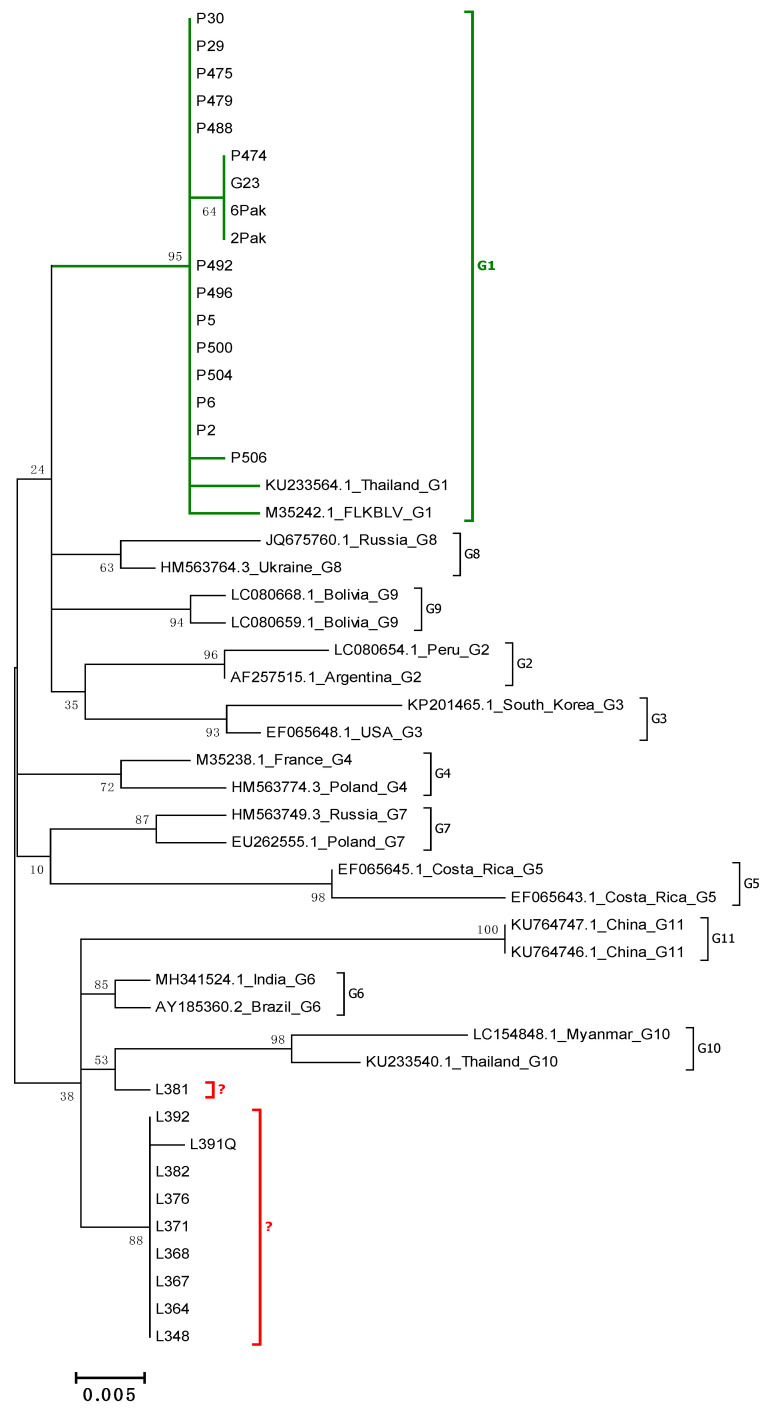
Phylogenetic analysis by the maximum likelihood method of a 400 bp fragment of *env* gene nucleotide sequences of Pakistan BLV isolates and known 11 BLV genotypes. The evolutionary history was inferred by using the maximum likelihood method based on the Kimura 2-parameter model. The percentage of trees in which the associated taxa clustered together is shown next to the branches. A discrete Gamma distribution was used to model evolutionary rate differences among sites. The tree is drawn to scale, with branch lengths measured in the number of substitutions per site. The analysis involved 48 nucleotide sequences. There was a total of 400 positions in the final dataset. Evolutionary analyses were conducted in MEGA6.

To determine the evolutionary relationship among BLV sequences from Pakistan, phylogenetic trees were constructed using maximum-likelihood method and *env* gene sequences, representing both lengths (400 bp and 903 bp). First, a phylogenetic tree was constructed using 26 sequences, 400 bp in length (and 22 sequences of BLV isolates representing all currently known genotypes G1–G11). Out of 26 sequences analyzed, 23 were retrieved from available sequences of 903 bp long, while the remaining three were directly generated from 444 bp amplicons. The reason to include the shorter sequences in the analysis was that these sequences have been used most often in phylogenetic analysis and they are exclusively available from the newly identified genotype G11. Furthermore, three Pakistani sequences, 2Pak, 6Pak and G23, were obtained only from shorter amplicons. All 26 sequences were clustered into two distinct groups (Figure 1). Seventeen of them were classified in genotype G1, which was supported by a high 95% bootstrap value. The remaining nine sequences clustered into an independent branch created by genotypes G6, G10 and G11, with a relatively low bootstrap value of 38%. Eight of them were grouped into one cluster closely related to the sequences representative for genotype G6, and one sequence (L381) was placed separately, but nearby genotype G10, which was supported by a bootstrap value of 53%. However clear genotype affiliation of these nine sequences was difficult based on such analysis.

**Figure 2:**
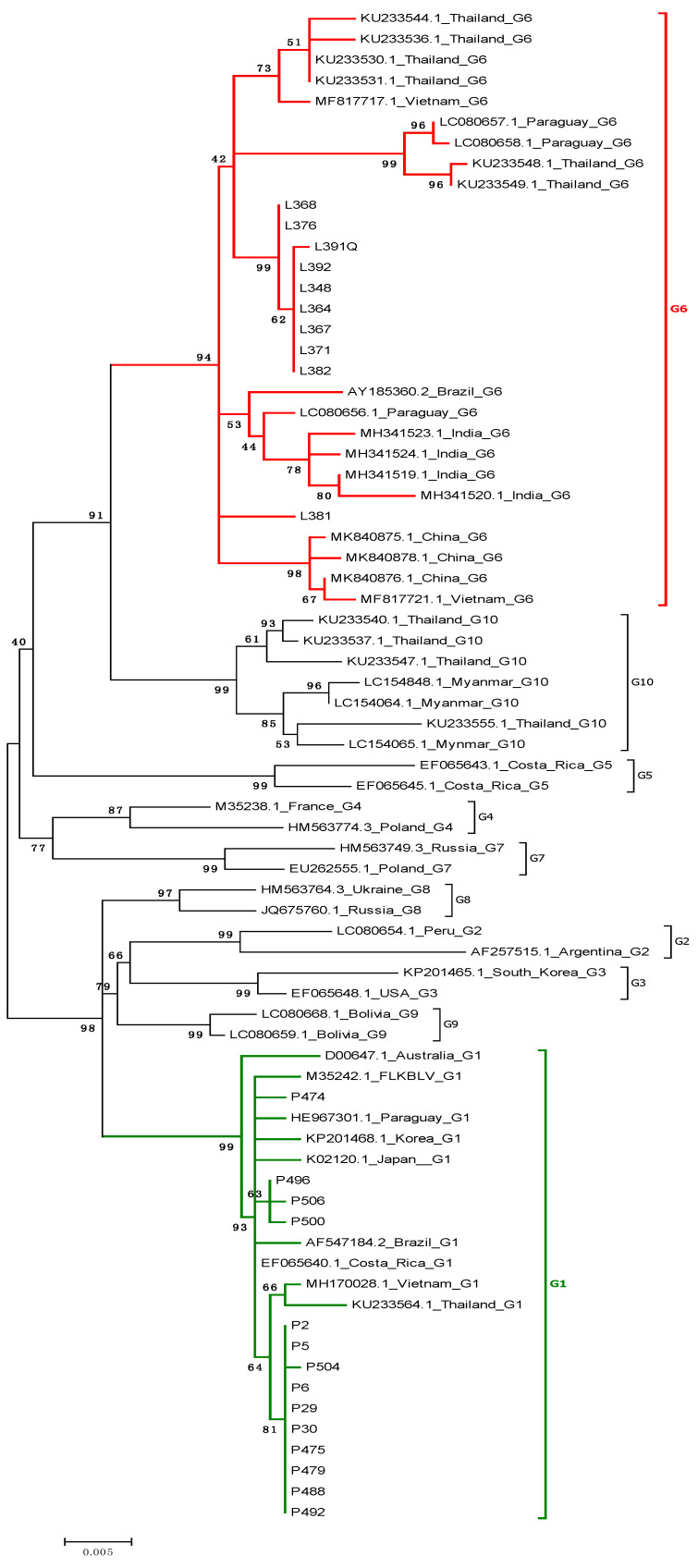
Phylogenetic analysis by the maximum likelihood method of the gp51 encoding 903 bp fragment of *env* gene sequences of Pakistan isolates. The evolutionary history was inferred by using the maximum likelihood method based on the Kimura 2-parameter model. A discrete Gamma distribution was used to model evolutionary rate differences among sites. The tree is drawn to scale, with branch lengths measured in the number of substitutions per site. The analysis involved 73 nucleotide sequences. There was a total of 903 positions in the final dataset. Evolutionary analyses were conducted in MEGA6.

To support the genotyping results based on the analysis of the 400 bp fragment, and to clear up inconclusive data on sequence clustering within genotype G6 24, sequences of 903 bp fragments were subjected to phylogenetic analysis. In addition, we included all 19 reference sequences representative for genotype G6. It was found that eight sequences, previously suspected to be affiliated with genotype G6, were clearly ranked in genotype G6, with a high bootstrap value 94%. Furthermore, phylogenetic analysis showed that these sequences could be segregated into at least two subgroups. In addition, one sequence, L381, previously suspected to be located in G10, now created a separate branch affiliated to genotype G6 (Figure 2). All remaining 14 sequences, as previously, belonged to genotype G1. The results of the phylogenetic analysis were strongly supported by estimation of evolutionary divergences (Table S2).

### 2.3. Analysis of Subtyping within Genotype G6

**Figure 3:**
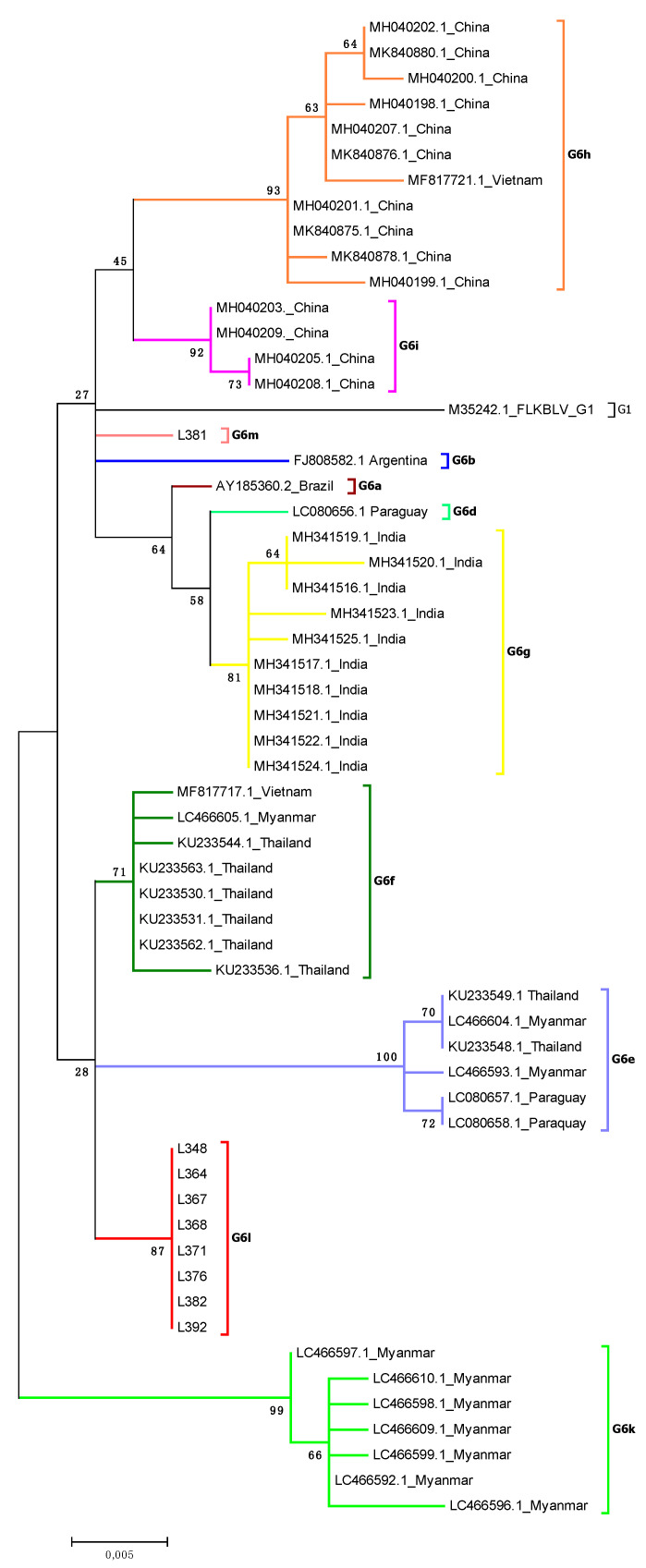
Phylogenetic analysis by maximum likelihood method of the gp51 encoding 501 bp fragment of *env* gene nucleotide sequences of BLV genotype 6 subgenotypes. The evolutionary history was inferred by using the maximum likelihood method based on the Kimura 2-parameter model. The percentage of trees in which the associated taxa clustered together is shown next to the branches. The tree is drawn to scale, with branch lengths measured in the number of substitutions per site. The analysis involved 59 nucleotide sequences. There was a total of 501 positions in the final dataset. Evolutionary analyses were conducted in MEGA6.

Since phylogenetic analysis based on 903 bp fragments provided the data on the existence of at least two subgenotypes within genotype G6, we attempted to classify possible subgenotypes in the G6 genotype by taking into account all the sequences known to date to be affiliated with this genotype. However, we found some inconsistency in classification of several sequences to a particular subgenotype of G6 in recently published data [30,42,43]. Most of sequences available from GeneBank and affiliated so far with genotype G6, had lengths shorter than 903 bp; therefore, this analysis included sequences of 501 bp length, which were previously used in other studies [43,44]. In addition, 40 available sequences 400 bp long, which contained molecular markers allowing their affiliation to particular subgenotype of the G6 genotype, were also considered in this analysis (Table S3). Additionally, chronological dates of the publishing of particular sequences were considered in the nomenclature of each subgenotype (Table S3). A newly proposed classification of subgenotype within genotype G6 is shown in Figure 3 and Table S3. This includes the existence of 13 subgenotypes, named from G6a to G6m. Eight sequences isolated from cattle from Pakistan belonged to subgenotype G6l, while one sequence, L381, was designed putative subgenotype G6m (Figure 3).

### 2.4. Comparison of Nucleotide Sequences Belonging to Genotype G1 and G6 

Pairwise comparison of 903 bp long sequences obtained from Pakistani cattle with 21 other sequences representative for genotype G1 from different countries and continents was carried out. The identity scores were represented as color-coded blocks using SDT v.1.2 software (Figure S1), and numerical values are showed in excel files (Table S4). The sequence identity among Pakistani sequences was more than 99.6%, since nine out of 14 sequences (P2, P5, P6, P29, P30, P475, P479, P488, P492) were identical. They showed the highest identity with sequences found in Costa Rica (99.8%) and Vietnam (99.7%). The similarity of the remaining five sequences was 99.4–99.9% and they were closely related to the sequences from Costa Rica (EF065640.1), Dominica (KX674372.1) and Uruguay (HE967301.1). When pairwise comparisons were extended over the three sequences 400 bp long, they indicated 100% identity to each other, and showed 99.8–100% similarity with sequences coming from Myanmar (LC466603.1), Costa Rica (EF065640.1) and Peru (LC075546.1) (Figure S2, Table S4).

**Figure 4:**
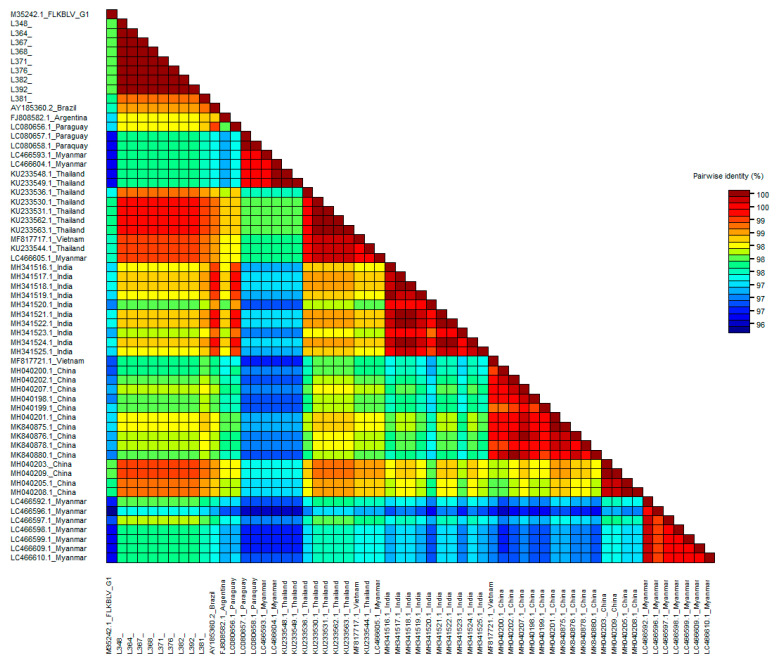
SDT color-coded matrix of pairwise identity scores generated by the alignment of a G6 501 bp long BLV *env* gene set of nucleotide sequences for nine Pakistan BLV isolates and 49 G1 representatives from other parts of the world.

As for genotype G1, pairwise identity of sequences classified within genotype G6 and 903 bp long, was calculated (Figures S3 and S5). Sequence identity of nine sequences representing Pakistani cattle ranged from 98.9 to 100%. The highest identity (98.8–99.1%) was noted with comparison to sequences from Thailand (KU233530.1, KU233531.1). The same relationship was noted when 30 additional sequences, 501 bp long, were included in the analysis. This clearly indicates the close relationship of sequences from Pakistan with Thailand, showing 98.8–99.4% identity (Figure 4 and Table S5). Furthermore, the analysis with 501 bp long sequences allowed clear confirmation of the existence of 13 different subgenotypes in the genotype G6, previously defined by the use of molecular markers and phylogenetic analysis (Table S3 and Figure 3).

### 2.5. Nucleotide and Amino Acid Sequence Analysis

Nucleotide variability was analyzed in 24 sequences of 903 bp length with respect to the functional determinants within the *env* gene (Figure S4). The alignment with the reference FLK-BLV sequence (M35242.1) revealed the presence of 45 mutations, of which seven and 33 were noticed exclusively in sequences representing genotype G1 and genotype G6, respectively. Five mutations were observed in sequences belonging to both G1 and G6 genotypes. Twelve out of 45 were nonsynonymous mutations A10G, C34G, G83C, G86A, G142A, A221G, T246T, T431C, C761T, C790T, G800A and G871A, leading to amino acid (aa) substitutions.

Alignment of gp51-deduced aa sequences with the FLK-BLV reference sequence (M35242.1), demonstrated that four substitutions (K4E, Q12E, C28S and R29Q) were localized at signal peptides, three (A48T, K74R and S82F) were found in epitope G, substitution I144T was seen in neutralizing domain 2 (ND2), three substitutions S254L, P264S and R267K were localized in the DD’ epitope and, finally, substitution A291T was found in epitope A (Table 2). This analysis showed the presence of substitutions specific for genotypes G1 and G6, as well as those typical for both G1 and G6 genotypes. C28S substitution, presented in 10 out of 14 sequences, and A294T substitution, found only in one sequence (P504), were specific for genotype G1. Out of nine amino acid substitutions found in genotype G6, the substitution P264S, present in six sequences, was found to be specific for sequences from Pakistan. The remaining aa changes included Q12E, R29Q, A48T, K74R, S82F, I144T, S254L substitutions. Although all these substitutions were found in sequences clustering within genotype G6, they were also classified within different genotypes when analyzed by other studies [29]. Therefore, they cannot be authoritatively classified as specific to genotype G6. Similarly, substitution R267K was seen in all nine G6 sequences, but was also frequently found in sequences typical for genotype 8, isolated from Russia (JQ675760.1, JQ675759.1) and Ukraine (HM563764.3). K4E substitution was found in all 24 Pakistani sequences, representing both G1 and G6 genotypes.

### 2.6. NGS Analysis of DNA from Sample L391

**Figure 5:**
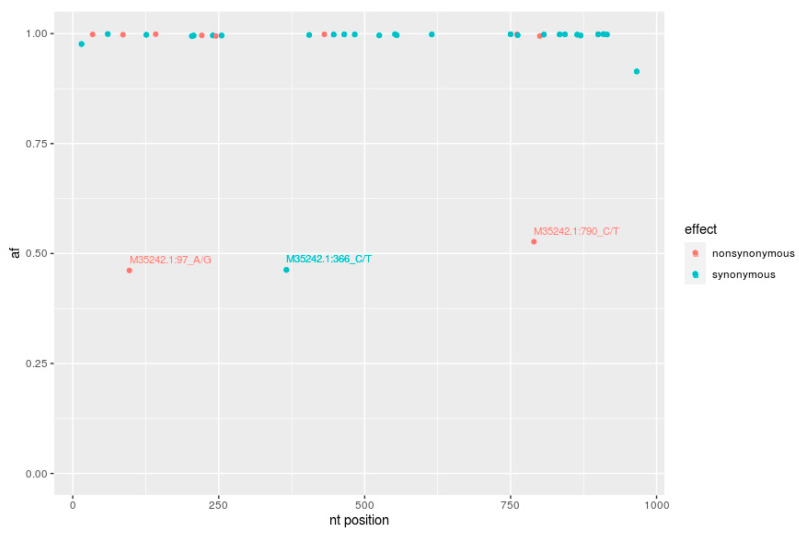
Frequency distribution of single nucleotide variants (SNVs) across the gp51 encoding 903 bp fragment of *env* gene sequences of an L391 sample. Synonymous (•) and nonsynonymous (•) SNVs identified after mapping trimmed reads to the reference BLV sequences (M35242.1_FLKBLV). Unique SNVs are described in detail (af—allele frequency, nt position—nucleotide position).

Careful analysis of the L391 sequence, 903 bp long, previously classified within genotype G6, revealed that there were two overlapping signal peaks in the respective electropherogram corresponding to the positions 97, 366 and 790. These peaks were predicted to encode transitions between A and G at the position 97 and C and T at both 366 and 790 positions. These nucleotide polymorphisms, seen when sequencing by the Sanger method, could suggest the circulation of a heterogeneous population of BLV proviruses in heifer L391. Thus, we decided to perform deep analysis of this sequence using NGS technology. The average number of reads for this sequence was 6852. Single nucleotide variants (SNVs) were analyzed to determine the intra-host variability of BLV and to identify genetic variants, known as quasispecies. SNV distribution is presented in Figure 5. Out of a total of 37 SNVs, 34 (89%) appeared with 99% frequency, and these mutations were equivalently found when Sanger sequencing was applied on sample L391. The remaining three mutations were exclusively noted in sequences generated by NGS, and they included mutations at position 97 (ACT→GCT, nonsynonymous T→A) with frequency 46%, position 366 (TTC→TTT, synonymous T→T) with frequency 46% and at position 790 (CCT→TCT, nonsynonymous, P→S) with frequency 53%. Regarding the localization of these changes, the mutation A→G was found in the codon corresponding to the last amino acid of the signal peptide. In fact, threonine (T) was substituted by alanine (A) at position 33 (T33A). The second mutation C→T was situated in the codon corresponding to amino acid 122, which precedes the first amino acid of the G epitope. Since this mutation was synonymous, it does not change any amino acid. The third mutation C→T was situated in the codon encoding aa 264 in epitope DD’, leading to substitution of phenylalanine (P) to serine (S).

## 3. Discussion

**Figure 6:**
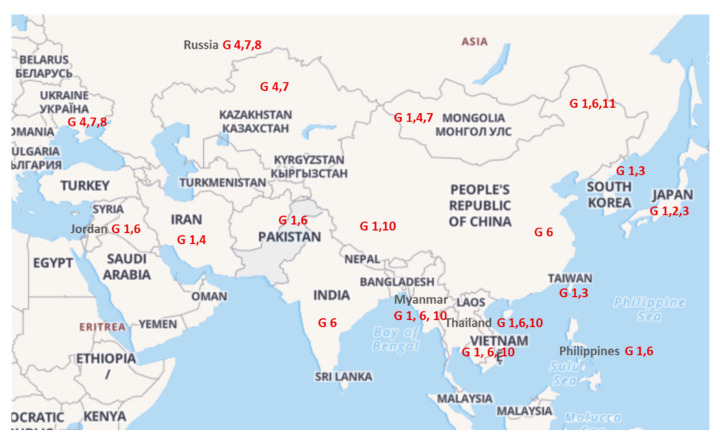
Distribution of BLV genotypes in Asia compiled from published literature. Map of Asia adapted from https://24timezones.com/worldmap#gref (accessed on 23 June 2021).

This study, showing the circulation of genotypes G1 and G6 in BLV-infected cattle from Pakistan, supports the general concept of geographically-tailored distribution of BLV genotypes, including Asiatic countries (Figure 6). According to previously published phylogenetic data, BLV isolates collected in countries belonging to the Asian continent are represented by worldwide distributed genotypes such as G1, G2, G3, G4, G5, G6, G7, G8, G10 and G11 [4,35]. When looking at the geographical context of these genotypes, it is clear that their dispersal is specifically associated with the northern and southern part of the Asian continent. While in the northern part the genotypes G4, G7 and G8 were exclusively identified in countries like Mongolia, Russia and Kazakhstan [26,45,46,47], in the southern part, genotypes G6 and G10 were found predominantly in China [35,48,49], Jordan [50], India [30], Myanmar [43], Thailand [27], Vietnam [42,51] and the Philippines [52]. In addition, other genotypes were associated with countries located at the southeastern part of Asia. Namely, BLV isolates originating from South Korea and Taiwan clustered in Genotype G3 [25,53], genotypes from Japan were identified within genotypes G1, G2 and G3 [54,55,56] and recently identified genotype G11 was characteristic for cattle from the northeast part of China [35]. This picture mirrors the geographical dispersal of BLV genotypes and points out the important role of cattle movement as a major driver of BLV transmission [43,57]. Indeed, the occurrence of genotypes G4, G7 and G8, typical for central and eastern European countries [26], in countries like Kazakhstan, Mongolia and the Asiatic part of Russia, results from cattle trade that took place between the former republics of the Soviet Union and countries belonging to the Council for Mutual Economic Assistance (CMEA) in the second half of the 20th century. Likewise, the appearance of the genotype G1 in the middle east and south and east Asiatic countries would be the result of cattle purchase, primarily from the USA, where infection with BLV was known to be present [7] and where genotype G1 was predominant [54].

Seventeen sequences from Pakistan were clustered to genotype G1, and their level of similarity ranged from 98.9–100% due to the fact that majority of them showed 100% nucleotide identity when both 400 bp and 903 bp long sequences were compared to each other. This was not surprising, since overall variability within genotype G1 is one of the lowest among other BLV genotypes [29]. Similarly, intragenotype mean nucleotide distance for genotype G1 was 0.0069, while the distances for genotypes G6 (0.0267) and G10 (0.0273) were prominently higher [29]. All sequences from Pakistan, classified in genotype G1, clustered together tightly with short branch lengths, and most of them were classified according to their place of origin. One can speculate that the tight phylogenetic clustering of G1 sequences from different locations could indicate their origin from a founder virus introduced in the past to the hosts in the particular region. This raises the question about the origin of BLV in Pakistan. As was explained by Rodriguez et al. [23], dispersion of BLV genotypes has been driven by worldwide human and animal migration and, undoubtedly, the purchase of cattle from infected herds is a risk factor in the BLV transmission [9]. Genotype G1 is distributed worldwide and is found in the US, South America, Asia and Australia. Thus, we can suppose that this genotype was transmitted and spread in Pakistan through the import of exotic cattle and bull semen from the countries where EBL was noted. A similar conclusion was recently made by Moe et al. [43] who analyzed the appearance of the genotype G1 in infected cattle in Myanmar. 

The existence of the genotype G6 has been reported in South American countries like Argentina, Brazil, Peru, Paraguay, Bolivia [23,24,34] and in Asia: Philippines [52], Thailand [27], India [30]. Taking into account the different length of *env* gene sequences, several studies revealed the existence of three [27,34,52] and four [30] subgenotypes within genotype G6. In the presented study, however, we noted some inconsistency in affiliation of sequences to the particular subgenotypes. The main reason for this was the consideration of too short fragments of the *env* gene in the phylogenetic tree. Indeed, the classification of sequences to a specific subgenotype depends on the fragment that was analyzed [30,42]. When longer, 903 bp, sequences were considered, all Pakistani sequences from genotype G6 fell into two subgenotypes. To upgrade the G6 subgenotyping, we wanted to include all sequences from the GeneBank affiliated so far with the genotype G6. Given that some of them represent fragments shorter than 903, a new analysis based on the 501 bp fragment was performed. The resulting tree segregated all these sequences into at least 11 subgenotypes, which was supported by respective high bootstrap values. This was justified not only by phylogenetic analysis, but also by the SDT matrix identity and by an identification of molecular markers approach, i.e., the methods recently described for subtyping of RSV [58]. Based on these new data sets, we proposed the reclassification of subgenotypes within genotype G6 with well distinguished, geographically related 13 subgenotypes, named G6a to G6m. This allocation revealed the existence of subgenotypes G6l and G6m, grouping the sequences exclusively from Pakistan. Similarly, subgenotypes G6g, G6i and G6k, encompassed the sequences only from India, China and Myanmar, respectively. Other subgenotypes, such as G6e, G6f and G6h, grouped the sequences from different countries such as Thailand-Myanmar-Paraguay, Vietnam-Myanmar-Thailand and China-Vietnam, respectively. These results are not surprising since common clustering of the sequences from Thailand, Myanmar and Paraguay was recently presented by Moe et al. [43]. This can be explained by epidemiological links existing between animals from these countries, resulting from the export of frozen semen and dairy cattle from Thailand to Myanmar [43] and the trade of cattle between neighboring countries like Vietnam, Myanmar, Thailand and China [42].

When predicted amino acid sequences were aligned to the FLK-BLV reference sequence with the annotation of epitopes and functional domains, twelve substitutions were noted. This reflects a relatively high degree of conservation of Pakistani sequences, which is consistent with more recent data showing a relatively low degree of BLV variability [4,54]. This analysis also revealed that all these substitutions were found in well-defined domains of the gp51 glycoprotein rather than at random locations, which is not surprising, since the similar accumulation of aa substitutions in BLV isolates from geographically distinct origins has been reported [29,34,43,59]. It is interesting that only one substitution out of the twelve reported in this study has not been described previously.

Four substitutions were located in the leader peptide. The envelope leader peptide is primarily responsible for the transport of polypeptide to the endoplasmic reticulum of infected cells and, therefore, plays an important role in incorporation of Env protein into nascent virions. It was shown that some mutations within the signal peptide of HIV-1 can be associated with viral infectivity [60]. The significance of aa changes reported in this study is unknown; however, it seems rather unlikely that these substitutions could affect biological functions of the leader peptide since similar changes have been found in other studies. These include the cattle exposed to BLV under natural conditions resulting in mounting of specific antibodies and the presence of proviral DNA [25,27,29,49,54]. Furthermore, substitutions K4E and R29Q found in BLV from LB59 cells cultured in vitro, led to the production of equal amounts of BLV as those seen for viruses harvested from FLK-BLV cells, which showed the conservation of lysine (K) and arginine (R) at positions 4 and 29, respectively [61].

Apart from changes seen in the signal peptide, three aa substitutions were localized within epitope G, which along with epitopes H and F is known as the conformational epitope involved in BLV infectivity, eliciting neutralizing antibodies and syncytia formation [17,62]. These epitopes located on the surface of the gp51 glycoprotein become accessible to neutralizing antibodies, which under strong positive selection lead to selection of BLV antigenic variants [18,61]. Our results reporting aa substitutions at residue 48, 74 and 82 are consistent with previous studies showing the presence of aa substitutions, mainly within epitopes G and H [26,29,54,59], which highlighted their role in viral escape strategy [18]. Despite the fact that several BLV variants lacking conformational epitopes have been identified in naturally infected animals, the knowledge on their impact on virus infectivity, disease progression and the host immune response is still limited. Although some studies have documented the existence of variants that evade the immune response [38,63], most of them were found in serologically positive cows. Therefore, it seems difficult to determine their real potential to circumvent the immune response, in particular to analyze whether they can affect the diagnostic capacity of serological tests in vitro. In our recent study we demonstrated that baculovirus expressed recombinant gp51 proteins lacking almost the entire epitope G or lacking a part of epitope G and whole epitope H, showed higher binding capacity to homologous sera in ELISA than to sera from animals infected with BLV expressing conserved gp51 epitopes [64]. This indicates that the epitope region of gp51 BLV determines the avidity of antigen/antibody reaction, and further study is needed to determine the possible effect of these amino acid variations on diminishing immunoreactivity of the conformational epitopes. This would potentially affect antibody detection by standard serological methods. This is of particular concern when blocking or competition ELISA, employing monoclonal conjugate directed to particular epitopes, is widely used.

Location of substitution I144T in the second neutralization domain (ND2) was in line with previous studies [23,26,28]; however, other aa changes at different residues were also reported in this domain [22,23,29]. Some substitutions can be exposed on the surface of ND2, possibly diminishing its immunoreactivity, as was shown by the use of 3D structural homology modelling of BLV gp51 [22]. It was also demonstrated that ND2 undergoes negative selection [59,65], which additionally underlines the importance of this domain in virus-host interaction. Amino acid substitutions were also found within two liner epitopes DD’ and A, located at the highly glycosylated C-terminal part of the gp51 protein [37]. Out of three substitutions within epitope DD’, the substitution S254L was frequently found in many others isolates [54]. These represented G2, G3, G4, G6, G6, G9 and G10 genotypes [29], while substitution P264S was typical for genotype G6 only. Epitope DD’ could be involved in the infectivity process, since substitution of tryptophan by arginine at position 261 (W261R) abolished syncytia formation with concurrent impairment of surface glycoprotein expression [15]. Since the aa substitutions found in this study were adjacent to residue 261, it can be speculated that they can be involved in the immunostimulatory or fusogenetic properties of epitope DD’. Single substitution A291T identified in epitope A was previously described in sequences from Costa Rica [54], and at the same residue 291 among the twelve Moldovan strains belonging to G7, a substitution of alanine to valine was indicated [59]. In addition, computational analysis of envelope glycoproteins from 256 divergent BLV isolates showed that this residue was the major site where positive selection occurs [66].

In this study, NGS was employed to analyze L391 sequences to estimate background levels of nucleotide polymorphisms of a 903 bp fragment of the *env* gene, since initial use of the Sanger method gave inconclusive results. A certain degree of heterogeneity at nucleotide and amino acid levels was observed. Despite the fact that 34 of the SNVs were dominant mutations that appeared with a frequency of 99%, and they were also detected by the Sanger method, another three SNVs were detected by NGS at a frequency level from 46% to 53%. The *env* gene of BLV is characterized by extremely low levels of intra-strain variability, as was documented in experimentally infected sheep [67]. In light of our results, it can be assumed that low degrees of intrahost *env* gene sequence variation can also exist in some individuals following natural BLV infection. Animal L391 was a six-month-old heifer, so it represented an early phase of BLV infection. This phase is characterized by horizontal spread of the virus and the generation of reverse transcriptase-associated substitution in provirus sequences, unlike the late stage of infection characterized by persistent infection, associated with clonal expansion of proviruses and somatic mutations [68]. Therefore, it seems convincing that nucleotide changes found in the L391 sequence were reverse transcriptase-dependent. Next to detection of diverse proviral sequences, coexisting within *env* gene, this study showed the potential existence of different antigenic forms of gp51, since the mutations at position 97 and 790 were nonsynonymous and led to aa substitutions. The circulation of a diverse form in one host can raise the key question whether such diversity would be maintained in nascent viruses or would select dominant viral variants. In addition, the observed BLV variants may indicate the presence of two closely related strains of BLV in the same infected animal or might suggest that BLV can form quasispecies. Therefore, more studies will be required to analyze the extent of intrastrain genetic variability at various time points after infection, using a larger group of infected cattle.

In conclusion, this study showed for the first time, phylogenetic analysis of the BLV *env* gene sequences in cattle from Pakistan, with their subsequent molecular analysis. These studies complement the major gap in BLV research, which is the lack of a sufficient number of available viral sequences representing locally circulating strains. Furthermore, these results shed new light on the degree of intrahost variability of BLV and may encourage further investigation in this direction.

## 4. Materials and Methods

### 4.1. Sample Collection and DNA Extraction

Whole blood samples were collected from 31 adult dairy cattle from four farms: 16 from farm H, one from each of the farms G1 and G2, and 13 from farm B, located in the Khyber Pakhtunkwa, Gilgit Baltisan and Punjab regions. All animals were serologically positive to BLV by ELISA [41]. In addition, nine tissue samples (from the spleen, lymph nodes and clotted blood) were collected from three animals located in farm H that were suspected of having clinical signs of EBL. Peripheral blood leukocytes (PBLs) were isolated by centrifugation at 1500× *g* for 25 min and erythrocytes were hemolyzed by osmotic shock with H2O and 4.5% NaCl. After two washes in PBS, the supernatant was discarded, and the cell dry pellets were sent together with tissue samples to NVRI in Puławy refrigerated with cooler packs. The genomic DNA was extracted using NucleoSpin Blood Kit and Nucleospin Tissue kit (Macherey Nagel GmbH & Co KG, Dueren, Germany), according to the manufacturer’s recommendation. The quality and quantity of DNA was evaluated in a Nanophotometer (Implen GmbH, Munich, Germany) and a bovine H3F3A housekeeping gene amplification was used to check the quality of DNA preparation [65,69].

### 4.2. PCR Amplification of 444 bp and 993 bp Fragments of env Gene

The amplification of a 444 bp fragment of the *env* gene was done by nested PCR as described by Fechner et al. [38] and recommended by the Manual of Diagnostic Tests and Vaccines for Terrestrial Animals [70]. Briefly, 500 ng of genomic DNA was amplified with the following cycling conditions: 2 min at 94 °C, 30 s at 95 °C, 30 s at 62 °C (*env* 5032 (5′-TCTGTGCCAAGTCTCCCAGATA-3′) (5032–5054); *env* 5608 (5′-AACAACAACCTCTGGGAAGGGT-3′) (5608–5630 or 30 s at 70 °C (*env* 5099 (5′-CCCACAAGGGCGGCGCCGG TTT-3′) (5099–5121), *env* 5521 (5′-GCGAGGCCGGGTCCA GAGCTGG-3′) (5521–5543), 1 min at 72 °C. After the last 40th cycle, the samples were incubated at 72 °C for 4 min. DreamTaq DNA Polymerase (2.5 units) (Thermo Scientific, Vilnius Lithuania) and respective buffer were added. After amplification, PCR products were separated and analyzed by electrophoresis on 1.5% agarose gel containing SimplySafe (EURX, Gdańsk, Poland) in 1x TAE buffer.

A seminested PCR was employed to amplify the 993 bp fragment of the *env* gene. PCR was performed using high-fidelity PrimeSTAR Max DNA Polymerase (Takara Bio, Kyoto, Japan) and forward primer AP_4762 (5′-GCTCTCCTGGCTACTGACC-3′) (4762–4780 [59] and reverse primers ZM2 (5′-TCTGATGGCTAAGGGCAGACACGGC-3′) (5786–5811) and ZM5 (5′-GCTAGGCCTAAGGTCAGGG CC GC-3′) (5733–5755) for nested PCR, respectively [71]. The reaction mixture contained 200 ng of DNA, 25 μL of PrimeSTAR Max DNA premix, 1 μL of each primer (2.5 μM) and 2.5 units of high-fidelity PrimeSTAR Max DNA Polymerase. For nested PCR, 5 μL of initial PCR mixture were used. For both PCRs, the same thermal conditions were used: 10 s at 98 °C, followed by 30 cycles of 10 s at 98 °C, 5 s at 55 °C and 10 s at 72 °C. After amplification, PCR products were separated and analyzed by electrophoresis on 1.5% agarose gel containing SimplySafe (EURX, Gdańsk, Poland) in 1x TAE buffer. Primer locations are given based on the BLV sequence (K02120).

### 4.3. DNA Sequencing and Sequence Analysis 

PCR products were purified using NucleoSpin Gel and PCR Clean-up (Macherey Nagel, GmbH & Co., Hamburg, Germany) and sequenced in both directions by the Genomed SA Company (Warsaw, Poland), using a 3730xlDNAAnalyzer (Applied Biosystems, Foster City, CA, USA) and a BigDye Terminator v3.1 Cycle Sequencing Kit. The sequence data were edited and aligned using the Geneious Alignment module within Geneious Pro 5.3 Software (Biomatters Ltd., Auckland, New Zealand). The resultant sequences representing the 400 bp and 903 bp fragments after subtracting the length of the primers were then submitted to the GenBank database and assigned accession numbers, as documented in Table S1. In addition, sequences representative for known genotypes G1-G11 were included in this analysis (Table S1). Phylogenetic analysis was conducted using MEGA 6 software [72]. The Kimura-2 parameter model with gamma distribution (K2 + G) was chosen as the model with the best fit for accurate phylogenetic analysis of 400 bp and 903 bp sequences using the “find best DNA/Protein models” tool of MEGA 6 software. The reliability of the phylogenetic relationships was evaluated by nonparametric bootstrap analysis with 1000 replicates. Estimation of evolutionary distances among sequences were conducted using the Kimura 2-parameter model (MEGA6). 

Subgenotype identification within genotype G6 was performed by phylogenetic analysis using MEGA 6 software (Kimura-2 parameter) and 501 bp long sequences retrieved from sequences generated in this study and sequences available from the GenBank data base (Table S1). Further identification of the existence of different subgenotypes of genotype G6 was carried out by adapting unique molecular markers analysis, described by Munoz-Escalante et al. [58]. All available genotype G6 sequences (July 2020) were aligned to each other, and all nucleotide changes were registered in comparison to the reference sequence (FLK-BLV M35242.1). This analysis was additionally enriched by 40 sequences 400 bp long (Table S1).

A pairwise identity matrix of sequences belonging to Genotype G1 (903 bp and 400 bp long sequences) and genotype G6 (903 bp and 501 bp long sequences) was inferred using Sequence Demarcation Tool Version 1.2 (SDTv1.2) software [73].

Deduction of amino acid sequences through translation of nucleotide to amino acid sequences was performed using BioEdit software [74].

### 4.4. Next-Generation Sequencing

A fragment of 903 bp of sequence L391 was subjected to NGS sequencing (Genomed SA, Warsaw) and the reaction was performed with an Illumina MiSeq instrument (Illumina, San Diego, CA, USA) using MiSeq Reagent Kit v3 (600-cycle) (Illumina, San Diego, CA, USA). The data were quality checked, trimmed using fastp [75] and mapped against reference sequence FLK-BLV (M35242) by bwa mem [76]. Next variant calling was done using a BBmap package [77], where the obtained variants were filtered and annotated by VariantAnnotation [78].

## Figures and Tables

**Table 1:** Bovine Leukemia Virus (BLV) detection results determined by PCR.

Sample ID	Origin of Sample	PCR Results ^1^	
		444 bp	993 bp
**J146 blood clot**	Khyber Pakhtunkwa (farm H)	-	-
J146 mesentery lymph node		-	-
J760 blood coat (**6Pak**)		**+**	-
J116 blood clot		-	-
J116 scapular lymph node		-	-
J116 lung lymph node (**2Pak**)		**+**	-
J116 liver		-	-
J116 spleen		-	-
J116 brain tissue		-	-
**P2**		+	**+**
P4		+	-
**P5**		+	**+**
**P6**		+	**+**
P7		+	-
**P29**		+	**+**
**P30**		+	**+**
**P474**		+	**+**
**P475**		+	**+**
**P479**		+	**+**
**P488**		+	**+**
**P492**		+	**+**
**P496**		+	**+**
**P500**		+	**+**
**P504**		+	**+**
**P506**		+	**+**
G6	Gilgit Baltisan (farm G1)	-	-
**G23**	Gilgit Baltisan (farm G2)	**+**	-
**L348**	Punjab (farm B)	+	**+**
L351		+	-
**L364**		+	**+**
**L367**		+	**+**
**L368**		+	**+**
**L371**		+	**+**
**L376**		+	**+**
****L381****		+	**+**
**L382**		+	**+**
L384		+	-
L387		+	-
**L391Q**		+	**+**
**L392**		+	**+**

^1^ Sample symbols with available sequences are in bold.

**Table 2:** Summary of amino acids substitution in the gp51 protein of 27 Pakistan BLV strains.

	Leader Peptide				G Epitope			Zn Binding Peptide				A
								ND2	D D’ Epitope			Epitope
	4	12	28	29	48	74	82	144	254	264	267	291
FLKBLV	K	Q	C	R	A	K	S	I	S	P	R	A
P5	E	.	S	.	.	.	.	.	.	.	.	.
P6	E	.	S	.	.	.	.	.	.	.	.	.
P479	E	.	S	.	.	.	.	.	.	.	.	.
P488	E	.	S	.	.	.	.	.	.	.	.	.
P492	E	.	S	.	.	.	.	.	.	.	.	.
P475	E	.	S	.	.	.	.	.	.	.	.	.
P2	E	.	S	.	.	.	.	.	.	.	.	.
P29	E	.	S	.	.	.	.	.	.	.	.	.
P30	E	.	S	.	.	.	.	.	.	.	.	.
P504	E	.	S	.	.	.	.	.	.	.	.	T
P474	E	.	.	.	.	.	.	.	.	.	.	.
P496	E	.	.	.	.	.	.	.	.	.	.	.
P500	E	.	.	.	.	.	.	.	.	.	.	.
P506	E	.	.	.	.	.	.	.	.	.	.	.
G23	~	~	~	~	~	~	~	.	~	~	~	~
2Pak	~	~	~	~	~	~	~	.	~	~	~	~
6Pak	~	~	~	~	~	~	~	.	~	~	~	~
L348	E	E	.	Q	T	R	F	T	L	S	K	.
L371	E	E	.	Q	T	R	F	T	L	S	K	.
L364	E	E	.	Q	T	R	F	T	L	S	K	.
L367	E	E	.	Q	T	R	F	T	L	S	K	.
L382	E	E	.	Q	T	R	F	T	L	S	K	.
L392	E	E	.	Q	T	R	F	T	L	S	K	.
L376	E	E	.	Q	T	R	F	T	L	.	K	.
L368	E	E	.	Q	T	R	F	T	L	.	K	.
L381	E	E	.	Q	T	R	F	T	L	.	K	.

BLV A strain substitution unique for Pakistan is highlighted in grey.

## Data Availability

The data presented in this study are openly available in GeneBank (for accession numbers see Table S1).

## Supplementary Materials

The following are available online at https://www.mdpi.com/article/10.3390/pathogens10070910/s1, Figure S1 SDT color-coded matrix of pairwise identity scores generated by the alignment of a G1 903 bp long BLV *env* gp51 set of nucleotide sequences for 14 Pakistan BLV isolates and 21 G1 representatives from other parts of the world. Figure S2 SDT color-coded matrix of pairwise identity scores generated by the alignment of a G1 400 bp long BLV *env* gene set of nucleotide sequences for 17 Pakistan BLV isolates and 35 G1 representatives from other parts of the world. Figure S3 SDT color-coded matrix of pairwise identity scores generated by the alignment of a G6 903 bp long BLV *env* gp51 set of nucleotide sequences for 9 Pakistan BLV isolates and 19 G6 representatives from other parts of the world. Figure S4: Summary of nucleotide substitution in the 903 bp fragment of *env* gene of 27 Pakistan BLV strains. Table S1: Identity and origin of the sequences analyzed in the study. Table S2: Estimates of evolutionary divergence between BLV sequences from Pakistan and other representatives worldwide. Table S3: Identification of molecular markers within Genotype 6 BLV sequences. Table S4: Pairwise identity scores of BLV sequences between G1 from Pakistan and other geographic regions worldwide. Table S5: Pairwise identity scores of BLV sequences between G6 from Pakistan and other geographic regions worldwide.
