# Supplementary material for: Molecular Characterization of the env Gene of Bovine Leukemia Virus in Cattle from Pakistan with NGS-Based Evidence of Virus Heterogeneity
Source: Pathogens. 2021 Jul 19;10(7):910. doi: 10.3390/pathogens10070910 (PMC8308526; doi:10.3390/pathogens10070910)
Supplement: Supplementary file 1 [file pathogens-10-00910-s001.zip › Table_S1_Identity and origin of the sequences analyzed in the study.pdf]

Table S1. Identity and origin of the sequences analysed in the study.

GenBank Accession No[1]	Name/Geographic origin	Genotype	Reference
MW934812	G23/Pakistan	1	This paper
MW934813	2Pak/Pakistan	1	This paper
MW934814	6Pak/Pakistan	1	This paper
MW926772	P2/Pakistan	1	This paper
MW926773	P5/Pakistan	1	This paper
MW926774	P6/Pakistan	1	This paper
MW926775	P29/Pakistan	1	This paper
MW926776	P30/Pakistan	1	This paper
MW926777	P474/Pakistan	1	This paper
MW926778	P475/Pakistan	1	This paper
MW926779	P479/Pakistan	1	This paper
MW926780	P488/Pakistan	1	This paper
MW926781	P492/Pakistan	1	This paper
MW926782	P496/Pakistan	1	This paper
MW926783	P500/Pakistan	1	This paper
MW926784	P504/Pakistan	1	This paper
MW926785	P506/Pakistan	1	This paper
MW926786	L348/Pakistan	6l	This paper
MW926787	L364/Pakistan	6l	This paper
MW926788	L367/Pakistan	6l	This paper
MW926789	L368/Pakistan	6l	This paper
MW926790	L371/Pakistan	6l	This paper
MW926791	L376/Pakistan	6l	This paper
MW926792	L381/Pakistan	6m	This paper
MW926793	L382/Pakistan	6l	This paper
MW926794	L392/Pakistan	6l	This paper
MZ148137	L391Q/Pakistan	6	This paper
PRJNA723743[1]	L391/Pakistan	nd	This paper
M35242.1	FLKBLV/USA	1	[1]
D00647.1	pBLV-A1/Australia	1	[2]
KU233564.1	Sk8-I2/Thailand	1	[3]
KJ668810.1	PCC67/Philippines	1	[4]
KJ668809.1	PCC130/Philippines	1	[4]
MH170028.1	S6F15/Vietnam	1	[5]
LC466601.1	KL16/Myanmar	1	[6]
LC466603.1	MC11/Myanmar	1	[6]
LC466594.1	HB13/Myanmar	1	[6]
LC466608.1	MW41/Myanmar	1	[6]
KP201468.1	GBGS-4/Korea	1	[7]
MN167087.1	2016NCHU017/Taiwan	1	[8]
MN167088.1	2016NCHU018/Taiwan	1	[8]
MN167089.1	2016NCHU019/Taiwan	1	[8]
K02120.1	Japan	1	[9]
AP018007.1	pvAF076/Japan	1	[10]
EF065661.1	JPMI-2 /Japan	1	[11]

LC060798.1	BLV_Mongolia-7/Mongolia	1	[12]
KY421037.1	E122/China	1	[13]
KU764753.1	E225/China	1	[13]
EU266060.1	Shahrekord/Iran	1	[14]
EF065640.1	CRAG-2/Costa_Rica	1	[11]
HE967301.1	LS1/Uruguay	1	[15]
LC080651.1	asun1/Paraguay	1	[16]
LC075546.1	Lima-93/Peru	1	[16]
LC080654.1	lima40/Peru	2	[16]
AF257515.1	Argentina	2	[17]
KP201465.1	GBGS-12/South_Korea	3	[7]
M35238.1	LB59/France	4	[1]
HM563774.3	68/Poland	4	[18]
EF065643.1	CRLV/Costa_Rica	5	[11]
EF065645.1	CRAG-1/Costa_Rica	5	[11]
AY185360.2	151/Brazil	6a	[19]
DQ059415.1	25/Brazil	6a	[20]
JN254639.1	CE08/180/SPBrazil	6a	[21]
GQ985389.1	10/Brazil	6a	Ikuno et al unpublished
LC075572.1	Montero-93/Bolivia	6a	[16]
LC075573.1	Montero-144/Bolivia	6a	[16]
LC075574.1	Montero-141/Bolivia	6a	[16]
LC075575.1	Bolivia_Ya-47/ Bolivia	6a	[16]
LC075553.1	Paraguray-98/Paraguay	6a	[16]
MH041958.1	COLAguachica130-9/Colombia	6a	[22]
MH041917.1	COLAguachica5251-7/Colombia	6a	[22]
MH041915.1	COLSotaquirGolondrina/Colombia	6a	[22]
MH041913.1	COLAguachica3994/Colombia	6a	[22]
FJ808582.1	PL-1238/Argentina	6b	[23]
JN254633.1	CE09/80/SP/Brazil	6b	[21]
GQ985391.1	29/Brazil	6b	Ikuno et al unpublished
KJ668815.1	PCC122/Philippines	6c	[4]
KJ668816.1	CAM69/Philippines	6c	[4]
KJ668817.1	PCC4/Philippines	6c	[4]
KJ668818.1	PCC158/Philippines	6c	[4]
KJ668819.1	PCC141/Philippines	6c	[4]
LC080656	par62/Paraguay	6d	[16]
LC080657.1	par89/Paraquay	6e	[16]
LC080658.1	par91/ Paraquay	6e	[16]
LC466593.1	HB9/Myanmar	6e	[6]
LC466604.1	MW18/ Myanmar	6e	[6]
KU233548.1	Pa53-A4/Thailand	6e	[3]
KU233549.1	Pa67-A5/ Thailand	6e	[3]
LC075549.1	Pucallpa-36/Peru	6e	[16]
LC075550.1	Pucallpa-187/Peru	6e	[16]
LC075551.1	Pucallpa-110/Peru	6e	[16]
LC075554.1	Paraguray-93/Paraguay	6e	[16]
[16]LC075552.1	Pucallpa-7/Peru	6e	[16]
LC075556.1	Paraguay-96/Paraguay	6e	[16]

LC075555.1	Paraguray-91 /Paraguay	6e	[16]
LC075571.1	Bolivia_Ya-102/Bolivia	6e	[16]
KU233536.1	Lo48-E4/Thailand	6f	[3]
KU233530.1	Ch17-F4/Thailand	6f	[3]
KU233531.1	Ch28-F5/Thailand	6f	[3]
KU233562.1	Sa11-H2/Thailand	6f	[3]
KU233563.1	Sa24-H3/Thailand	6f	[3]
MF817717.1	VT-S9/Vietnam	6f	[5]
KU233544.1	Ns94-D4/ Thailand	6f	[3]
LC466605.1	MW22/Myanmar	6f	[6]
MH341516.1	Ind_UK_251/India	6g	[24]
MH341517.1	Ind_UK_287/India	6g	[24]
MH341518.1	Ind_UK_237/India	6g	[24]
MH341519.1	Ind_UK_258/India	6g	[24]
MH341520.1	Ind_UK_283/India	6g	[24]
MH341521.1	Ind_UK_265/India	6g	[24]
MH341522.1	Ind_UK_250/India	6g	[24]
MH341523.1	Ind_UK_297/India	6g	[24]
MH341524.1	Ind_UK_264/India	6g	[24]
MH341525.1	Ind_UK_285/India	6g	[24]
MF817721.1	BV-S39/Vietnam	6h	[5]
MH040200.1	B1021/China	6h	[25]
MH040202.1	B1219/China	6h	[25]
MH040207.1	B1686/China	6h	[25]
MH040198.1	B628/China	6h	[25]
MH040199.1	B665/China	6h	[25]
MH040201.1	B1117/China	6h	[25]
MK840875.1	B3026/China	6h	Chen et al unpublished
MK840876.1	B3054/China	6h	Chen et al unpublished
MK840878.1	B3065/China	6h	Chen et al unpublished
MK840880.1	B3103/China	6h	Chen et al unpublished
KU764759.1	E318/ China	6h	[13]
KU764760.1	E325/ China	6h	[13]
KU764757.1	E312/ China	6h	[13]
KU764758.1	E3162/ China	6h	[13]
KU764748.1	E110/ China	6h	[13]
KU764749.1	E112/ China	6h	[13]
KU764750.1	E119/ China	6h	[13]
KU764756.1	E307/ China	6h	[13]
MH040203.1	B1340/China	6i	[25]
MH040209.1	B1833/China	6i	[25]
MH040205.1	B1405/China	6i	[25]
MH040208.1	B1831/China	6i	[25]
KU764751.1	E158/China	6i	[13]
LT970922.1	13287_2013/Italy	6j	[26]
LT970923.1	16661_2013/Italy	6j	[26]
LT970921.1	11327_2013/Italy	6j	[26]
LT970937.1	FV_2015/Italy	6j	[26]
LC466592.1_	HB8/ Myanmar	6k	[6]
LC466596.1	HT2/ Myanmar	6k	[6]

LC466597.1	HT11/ Myanmar	6k	[6]
LC466598.1	HT12/ Myanmar	6k	[6]
LC466599.1	HT17/ Myanmar	6k	[6]
LC466609.1	TA3/ Myanmar	6k	[6]
LC466610.1	TA8/ Myanmar	6k	[6]
HM563749.3	5/Russia	7	[18]
EU262555.2	151/Poland	7	[27]
HM563764.3	4-6/Ukraine	8	[18]
JQ675760.1	pr72/Russia	8	Lomakina et al unpublished
LC080668.1	por28/Bolivia	9	[16]
LC080659.1	mon1/Bolivia	9	[16]
LC154848.1	L1/ Myanmar	10	[28]
KU233540.1	ML45-B3/ Thailand	10	[3]
KU233537.1	Lo50-E5// Thailand	10	[3]
KU233547.1	Pa51-A3/Thailand	10	[3]
KU233555.1	Pr67-C5/Thailand	10	[3]
LC154064.1	L2/ Myanmar	10	[28]
LC154065.1	S5/ Myanmar	10	[28]
KU764746.1	E101/China	11	[3]
KU764747.1	E102/China	11	[3]

[1] In the case of an NGS result, this is the BIOPROJECT number.

References:

1. Mamoun, R.Z.; Morisson, M.; Rebeyrotte, N.; Busetta, B.; Couez, D.; Kettmann, R.; Hospital, M.; Guillemain, B. Sequence variability of bovine leukemia virus env gene and its relevance to the structure and antigenicity of the glycoproteins. *J. Virol.* **1990**, *64*, 4180–4188, doi:10.1128/jvi.64.9.4180-4188.1990.
2. Coulston, J.; Naif, H.; Brandon, R.; Kumar, S.; Khan, S.; Daniel, R.C.W.; Lavin, M.F. Molecular Cloning and Sequencing of an Australian Isolate of Proviral Bovine Leukaemia Virus DNA: Comparison with other Isolates. *J. Gen. Virol.* **1990**, *71*, 1737–1746, doi:10.1099/0022-1317-71-8-1737.
3. Lee, E.; Kim, E.-J.; Ratthanophart, J.; Vitoonpong, R.; Kim, B.-H.; Cho, I.-S.; Song, J.-Y.; Lee, K.-K.; Shin, Y.-K. Molecular epidemiological and serological studies of bovine leukemia virus (BLV) infection in Thailand cattle. *Infect. Genet. Evol.* **2016**, *41*, 245–254, doi:10.1016/j.meegid.2016.04.010.
4. Polat, M.; Ohno, A.; Takeshima, S.; Kim, J.; Kikuya, M.; Matsumoto, Y.; Mingala, C.N.; Onuma, M.; Aida, Y. Detection and molecular characterization of bovine leukemia virus in Philippine cattle. *Arch. Virol.* **2015**, *160*, 285–296, doi:10.1007/s00705-014-2280-3.
5. Dao, T.D.; Bui, V.N.; Omatsu, T.; Katayama, Y.; Mizutani, T.; Ogawa, H.; Imai, K. Application of the SureSelect target enrichment system for next-generation sequencing to obtain the complete genome sequence of bovine leukemia virus. *Arch. Virol.* **2018**, *163*, 3155–3159, doi:10.1007/s00705-018-3957-9.
6. Moe, K.K.; Polat, M.; Borjigin, L.; Matsuura, R.; Hein, S.T.; Moe, H.H.; Aida, Y. New evidence of bovine leukemia virus circulating in Myanmar cattle through epidemiological and molecular characterization. *PLoS One* **2020**, *15*, e0229126, doi:10.1371/journal.pone.0229126.
7. Lee, E.; Kim, E.-J.; Joung, H.-K.; Kim, B.-H.; Song, J.-Y.; Cho, I.-S.; Lee, K.-K.; Shin, Y.-K. Sequencing and phylogenetic analysis of the gp51 gene from Korean bovine leukemia virus isolates. *Virol. J.* **2015**, *12*, 64, doi:10.1186/s12985-015-0286-4.
8. Hsieh, J.-C.; Li, C.-Y.; Hsu, W.-L.; Chuang, S.-T. Molecular Epidemiological and Serological Studies of Bovine Leukemia Virus in Taiwan Dairy Cattle. *Front. Vet. Sci.* **2019**, *6*, doi:10.3389/fvets.2019.00427.

9. Sagata, N.; Yasunaga, T.; Tsuzuku-Kawamura, J.; Ohishi, K.; Ogawa, Y.; Ikawa, Y. Complete nucleotide sequence of the genome of bovine leukemia virus: its evolutionary relationship to other retroviruses. *Proc. Natl. Acad. Sci.* **1985**, *82*, 677–681, doi:10.1073/pnas.82.3.677.
10. Murakami, H.; Uchiyama, J.; Suzuki, C.; Nikaido, S.; Shibuya, K.; Sato, R.; Maeda, Y.; Tomioka, M.; Takeshima, S.; Kato, H.; et al. Variations in the viral genome and biological properties of bovine leukemia virus wild-type strains. *Virus Res.* **2018**, *253*, 103–111, doi:10.1016/j.virusres.2018.06.005.
11. Zhao, X.; Buehring, G.C. Natural genetic variations in bovine leukemia virus envelope gene: Possible effects of selection and escape. *Virology* **2007**, *366*, 150–165, doi:10.1016/j.virol.2007.03.058.
12. Ochirkhuu, N.; Konnai, S.; Odbileg, R.; Nishimori, A.; Okagawa, T.; Murata, S.; Ohashi, K. Detection of bovine leukemia virus and identification of its genotype in Mongolian cattle. *Arch. Virol.* **2016**, *161*, 985–991, doi:10.1007/s00705-015-2676-8.
13. Yu, C.; Wang, X.; Zhou, Y.; Wang, Y.; Zhang, X.; Zheng, Y. Genotyping bovine leukemia virus in dairy cattle of Heilongjiang, northeastern China. *BMC Vet. Res.* **2019**, *15*, 179, doi:10.1186/s12917-019-1863-3.
14. Hemmatzadeh, F. Sequencing and Phylogenetic Analysis of gp51 Gene of Bovine Leukaemia Virus in Iranian Isolates. *Vet. Res. Commun.* **2007**, *31*, 783–789, doi:10.1007/s11259-007-0012-9.
15. Moratorio, G.; Obal, G.; Dubra, A.; Correa, A.; Bianchi, S.; Buschiazzo, A.; Cristina, J.; Pritsch, O. Phylogenetic analysis of bovine leukemia viruses isolated in South America reveals diversification in seven distinct genotypes. *Arch. Virol.* **2010**, *155*, 481–489, doi:10.1007/s00705-010-0606-3.
16. Polat, M.; Takeshima, S.; Hosomichi, K.; Kim, J.; Miyasaka, T.; Yamada, K.; Arainga, M.; Murakami, T.; Matsumoto, Y.; de la Barra Diaz, V.; et al. A new genotype of bovine leukemia virus in South America identified by NGS-based whole genome sequencing and molecular evolutionary genetic analysis. *Retrovirology* **2016**, *13*, 4, doi:10.1186/s12977-016-0239-z.
17. Dube, S.; Dolcini, G.; Abbott, L.; Mehta, S.; Dube, D.; Gutierrez, S.; Ceriani, C.; Esteban, E.; Ferrer, J.; Poiesz, B. The Complete Genomic Sequence of a BLV Strain from a Holstein Cow from Argentina. *Virology* **2000**, *277*, 379–386, doi:10.1006/viro.2000.0622.
18. Rola-Łuszczak, M.; Pluta, A.; Olech, M.; Donnik, I.; Petropavlovskiy, M. The Molecular Characterization of Bovine Leukaemia Virus Isolates from Eastern Europe and Siberia and Its Impact on Phylogeny. *PLoS One* **2013**, *8*, 58705, doi:10.1371/journal.pone.0058705.
19. Camargos, M.F.; Pereda, A.; Stancek, D.; Rocha, M.A.; Reis, J.K.P. dos; Greiser-Wilke, I.; Leite, R.C. Molecular characterization of the env gene from Brazilian field isolates of Bovine Leukemia Virus. *Virus Genes* **2007**, *34*, 343–350, doi:10.1007/s11262-006-0011-x.
20. Gregory, L.; Carrillo Gaeta, N.; Araújo, J.; Matsumiya Thomazelli, L.; Harakawa, R.; Ikuno, A.A.; Hiromi Okuda, L.; de Stefano, E.; Pituco, E.M. Bovine leukaemia virus genotypes 5 and 6 are circulating in cattle from the state of São Paulo, Brazil. *J. Med. Microbiol.* **2017**, *66*, 1790–1797, doi:10.1099/jmm.0.000639.
21. D′Angelino, R.H.R.; Pituco, E.M.; Villalobos, E.M.C.; Harakava, R.; Gregori, F.; Del Fava, C. Detection of Bovine Leukemia Virus in Brains of Cattle with a Neurological Syndrome: Pathological and Molecular Studies. *Biomed Res. Int.* **2013**, *2013*, 1–6, doi:10.1155/2013/425646.
22. Corredor-Figueroa, A.P.; Salas, S.; Olaya-Galán, N.N.; Quintero, J.S.; Fajardo, Á.; Soñora, M.; Moreno, P.; Cristina, J.; Sánchez, A.; Tobón, J.; et al. Prevalence and molecular epidemiology of bovine leukemia virus in Colombian cattle. *Infect. Genet. Evol.* **2020**, *80*, 104171, doi:10.1016/j.meegid.2020.104171.
23. Rodriguez, S.M.; Golemba, M.D.; Campos, R.H.; Trono, K.; Jones, L.R. Bovine leukemia virus can be classified into seven genotypes: evidence for the existence of two novel clades. *J. Gen. Virol.* **2009**, *90*, 2788–2797, doi:10.1099/vir.0.011791-0.
24. Gautam, S.; Mishra, N.; Kalaiyarasu, S.; Jhade, S.K.; Sood, R. Molecular Characterization of Bovine Leukaemia Virus (BLV) Strains Reveals Existence of Genotype 6 in Cattle in India with

evidence of a new subgenotype. *Transbound. Emerg. Dis.* **2018**, *65*, 1968–1978, doi:10.1111/tbed.12979.
25. Yang, Y.; Chu, S.; Shang, S.; Yang, Z.; Wang, C. Short communication: Genotyping and single nucleotide polymorphism analysis of bovine leukemia virus in Chinese dairy cattle. *J. Dairy Sci.* **2019**, *102*, 3469–3473, doi:10.3168/jds.2018-15481.
26. Bazzucchi, M.; Iscaro, C.; Casciari, C.; Giammarioli, M.; Feliziani, F. Molecular characterization of Italian bovine leukemia virus isolates reveals the presence of distinct phylogenetic clusters. *Arch. Virol.* **2019**, *164*, 1697–1703, doi:10.1007/s00705-019-04255-4.
27. Pluta, A.; Rola-Łuszczak, M.; Kubiś, P.; Balov, S.; Moskalik, R.; Choudhury, B.; Kuźmak, J. Molecular characterization of bovine leukemia virus from Moldovan dairy cattle. *Arch. Virol.* **2017**, *162*, 1563–1576, doi:10.1007/s00705-017-3241-4.
28. Polat, M.; Moe, H.H.; Shimogiri, T.; Moe, K.K.; Takeshima, S.; Aida, Y. The molecular epidemiological study of bovine leukemia virus infection in Myanmar cattle. *Arch. Virol.* **2017**, *162*, 425–437, doi:10.1007/s00705-016-3118-y.
