# Supplementary material for: Molecular Characterization of the env Gene of Bovine Leukemia Virus in Cattle from Pakistan with NGS-Based Evidence of Virus Heterogeneity
Source: Pathogens. 2021 Jul 19;10(7):910. doi: 10.3390/pathogens10070910 (PMC8308526; doi:10.3390/pathogens10070910)
Supplement: Supplementary file 1 [file pathogens-10-00910-s001.zip › Figure_S1_Pairwise_identity_G1_903.pdf]

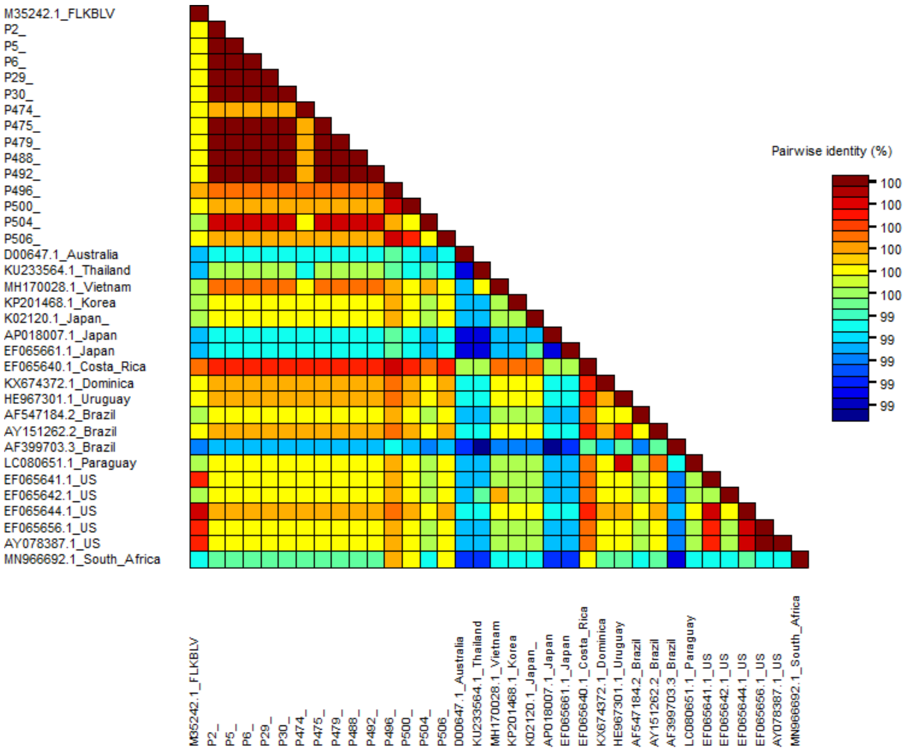

Figure S1. SDT colour-coded matrix of pairwise identity scores generated by the alignment of a G1 903 bp long BLV *env* gp51 set of nucleotide sequences for 14 Pakistan BLV isolates and 21 G1 representatives from other parts of the world.
