# Supplementary material for: Molecular Characterization of the env Gene of Bovine Leukemia Virus in Cattle from Pakistan with NGS-Based Evidence of Virus Heterogeneity
Source: Pathogens. 2021 Jul 19;10(7):910. doi: 10.3390/pathogens10070910 (PMC8308526; doi:10.3390/pathogens10070910)
Supplement: Supplementary file 1 [file pathogens-10-00910-s001.zip › Figure_S4 Summary of nucleotide_changes_Pakistan.pdf]

	6	10	15	34	60	83	86	87	126	142	165	204	207	221	240	245	246	255	405	431	447	465	483
Region	signal peptide									G				G			G		ND2			CD 8 T	
Epitope														CD8 T-cell(N5)				CD4	CD8 T (N11)	Zn-binding peptide; CD8 T (N12)			
FLKBLV	T	A	A	C	C	G	G	G	C	G	T	G	A	A	G	C	T	C	A	T	T	T	G
P5	.	G	.	.	.	C	.	.	.	.	C	.	.	.	.	.	.	.	.	.	C	.	.
P6	.	G	.	.	.	C	.	.	.	.	C	.	.	.	.	.	.	.	.	.	C	.	.
P479	.	G	.	.	.	C	.	.	.	.	C	.	.	.	.	.	.	.	.	.	C	.	.
P488	.	G	.	.	.	C	.	.	.	.	C	.	.	.	.	.	.	.	.	.	C	.	.
P492	.	G	.	.	.	C	.	.	.	.	C	.	.	.	.	.	.	.	.	.	C	.	.
P475	.	G	.	.	.	C	.	.	.	.	C	.	.	.	.	.	.	.	.	.	C	.	.
P2	.	G	.	.	.	C	.	.	.	.	C	.	.	.	.	.	.	.	.	.	C	.	.
P29	.	G	.	.	.	C	.	.	.	.	C	.	.	.	.	.	.	.	.	.	C	.	.
P30	.	G	.	.	.	C	.	.	.	.	C	.	.	.	.	.	.	.	.	.	C	.	.
P504	.	G	.	.	.	C	.	.	.	.	C	.	.	.	.	.	.	.	.	.	C	.	.
P474	.	G	.	.	.	.	.	.	.	.	.	.	.	.	.	.	G	.	.	.	C	.	.
P496	.	G	.	.	.	.	.	C	.	.	.	.	.	.	.	.	.	.	.	.	C	.	.
P500	.	G	.	.	.	.	.	C	.	.	.	.	.	.	.	.	.	.	.	.	C	.	.
P506	.	G	.	.	.	.	.	C	.	.	.	.	.	.	.	.	.	.	.	.	C	.	.
G23	~	~	~	~	~	~	~	~	~	~	~	~	~	~	~	~	~	~	.	.	C	.	.
2Pak	~	~	~	~	~	~	~	~	~	~	~	~	~	~	~	~	~	~	.	.	C	.	.
6Pak	~	~	~	~	~	~	~	~	~	~	~	~	~	~	~	~	~	~	.	.	C	.	.
L348	C	G	G	G	T	.	A	.	T	A	.	A	G	G	A	T	.	T	G	C	C	C	A
L371	C	G	G	G	T	.	A	.	T	A	.	A	G	G	A	T	.	T	G	C	C	C	A
L364	C	G	G	G	T	.	A	.	T	A	.	A	G	G	A	T	.	T	G	C	C	C	A
L367	C	G	G	G	T	.	A	.	T	A	.	A	G	G	A	T	.	T	G	C	C	C	A
L382	C	G	G	G	T	.	A	.	T	A	.	A	G	G	A	T	.	T	G	C	C	C	A
L392	C	G	G	G	T	.	A	.	T	A	.	A	G	G	A	T	.	T	G	C	C	C	A
L376	C	G	G	G	T	.	A	.	T	A	.	A	G	G	A	T	.	T	G	C	C	C	A
L368	C	G	G	G	T	.	A	.	T	A	.	A	G	G	A	T	.	T	G	C	C	C	A
L381	C	G	.	G	T	.	A	.	.	A	.	.	G	G	A	T	.	T	G	C	C	.	A

	492	525	552	555	561	606	609	615	750	760	761	790	800	807	816	834	843	864	870	871	876	900
Region										D and D' epitope				Transmembrane hydrophobic region								
Epitope		E epitope																A epitope				
FLKBLV	A	G	T	C	G	A	C	A	C	T	C	C	G	G	T	G	T	C	G	G	C	A
P5	.	.	.	.	.	.	T	.	.	T	.	.	.	.	.	.	.	.	.	.	.	.
P6	.	.	.	.	.	.	T	.	.	T	.	.	.	.	.	.	.	.	.	.	.	.
P479	.	.	.	.	.	.	T	.	.	T	.	.	.	.	.	.	.	.	.	.	.	.
P488	.	.	.	.	.	.	T	.	.	T	.	.	.	.	.	.	.	.	.	.	.	.
P492	.	.	.	.	.	.	T	.	.	T	.	.	.	.	.	.	.	.	.	.	.	.
P475	.	.	.	.	.	.	T	.	.	T	.	.	.	.	.	.	.	.	.	.	.	.
P2	.	.	.	.	.	.	T	.	.	T	.	.	.	.	.	.	.	.	.	.	.	.
P29	.	.	.	.	.	.	T	.	.	T	.	.	.	.	.	.	.	.	.	.	.	.
P30	.	.	.	.	.	.	T	.	.	T	.	.	.	.	.	.	.	.	.	.	.	.
P504	.	.	.	.	.	.	T	.	.	T	.	.	.	.	.	.	.	.	.	A	.	.
P474	.	.	.	.	.	G	T	.	.	T	.	.	.	.	.	.	.	.	.	.	.	.
P496	.	.	.	.	.	.	T	.	.	T	.	.	.	.	.	.	.	.	.	.	.	.
P500	.	.	.	.	.	.	T	.	.	T	.	.	.	.	.	.	.	.	.	.	T	.
P506	.	.	.	.	A	.	T	.	.	T	.	.	.	.	.	.	.	.	.	.	.	.
G23	.	.	.	.	.	G	T	.	~	~	~	~	~	~	~	~	~	~	~	~	~	~
2Pak	.	.	.	.	.	G	T	.	~	~	~	~	~	~	~	~	~	~	~	~	~	~
6Pak	.	.	.	.	.	G	T	.	~	~	~	~	~	~	~	~	~	~	~	~	~	~
L348	.	A	C	T	.	.	.	G	T	T	T	T	A	A	.	A	C	T	A	.	.	G
L371	.	A	C	T	.	.	.	G	T	T	T	T	A	A	.	A	C	T	A	.	.	G
L364	.	A	C	T	.	.	.	G	T	T	T	T	A	A	.	A	C	T	A	.	.	G
L367	.	A	C	T	.	.	.	G	T	T	T	T	A	A	.	A	C	T	A	.	.	G
L382	.	A	C	T	.	.	.	G	T	T	T	T	A	A	.	A	C	T	A	.	.	G
L392	.	A	C	T	.	.	.	G	T	T	T	T	A	A	.	A	C	T	A	.	.	G
L376	.	A	C	T	.	.	.	G	T	T	T	.	A	A	.	A	C	T	A	.	.	G
L368	.	A	C	T	.	.	.	G	T	T	T	.	A	A	.	A	C	T	A	.	.	G
L381	G	A	C	T	A	.	T	G	.	T	T	.	A	A	C	A	C	T	A	.	.	G

Figure S1. Summary of nucleotide substitution in the 903 bp fragment of env gene of 27 Pakistan BLV strains. Mutations characteristic for Genotype 1 are in red font, for genotype 6 - black font, and observed at both genotypes - green font. Nonsynonymous mutations are highlighted in orange
